# Simple hypertrophic tonsils have more active innate immune and inflammatory responses than hypertrophic tonsils with recurrent inflammation in children

**DOI:** 10.1186/s40463-020-00428-3

**Published:** 2020-06-01

**Authors:** Qun Huang, Hu Hua, Wei Li, Xi Chen, Lei Cheng

**Affiliations:** 1 Department of Otorhinolaryngology, Children’s Hospital of Nanjing Medical University, Nanjing, China; 2 Nanjing Key Laboratory of Pediatrics, Children’s Hospital of Nanjing Medical University, Nanjing, China; 3 Department of Otorhinolaryngology, The First Affiliated Hospital, Nanjing Medical University, 300 Guangzhou Road, Nanjing, 210029 Jiangsu China

**Keywords:** Palatine tonsil, Hypertrophy, Tonsillitis, Innate immunity, Inflammation, Infection

## Abstract

**Background:**

Tonsil hypertrophy has negative impact on children’s health, but its pathogenesis remains obscure despite the fact that numerous bacteriological studies have been carried out. Understanding the innate immune and inflammatory states of hypertrophic tonsils with different clinical manifestations is of great significance for defining the pathogenesis of tonsil hypertrophy and establishing treatment strategies. The present study was undertaken to examine the characteristics of innate immunity and inflammation in children with hypertrophic palatine tonsils and different clinical manifestations.

**Methods:**

Tonsil tissues were surgically removed from the patients and classified based on the patients’ clinical manifestations. The patients were divided into three groups: 1) Control group; 2) Tonsil Hypertrophy (TH) group; and 3) Tonsil Hypertrophy combined with Recurrent Infection (TH + RI) group. The immune and inflammatory statuses of these tissues were characterized using qRT-PCR and ELISA methods.

**Results:**

Viral protein 1 (VP1) was highly expressed in TH group, but not in TH + RI group. In TH group, elevated expression was observed in the innate immune mediators, including retinoic acid-inducible gene I (RIG-I), interferon alpha (IFN-α), mitochondrial antiviral-signaling protein (MAVS), NLR family pyrin domain containing 3 (NLRP3), toll-like receptor (TLR) 4 and TLR7. Consistent with the innate immune profile, the expression of inflammatory markers (IL-1β, NF-κB and IL-7) was also significantly elevated in TH group. Meanwhile, the COX-2/PGE2/EP4 signaling pathway was found to be involved in the inflammatory response and the formation of fibroblasts.

**Conclusions:**

Innate immune and inflammatory responses are more active in simple hypertrophic tonsils, rather than hypertrophic tonsils with recurrent inflammation. A local relative immune deficiency in the hypertrophic tonsils may be a causative factor for recurrent tonsillitis in TH + RI. These differences, together with the patient’s clinical manifestations, suggest that tonsillar hypertrophy might be regulated by diverse immune and/or inflammatory mechanism through which novel therapeutic strategies might be created.

## Background

The tonsil is an independent organ constructed by mucosal-associated lymphoid tissue (MALT). As the first stronghold against foreign pathogens, the tonsil can selectively regulate immune response in the internal environment [1, 2]. Simple tonsil hypertrophy refers to the enlargement of the tonsil uncomplicated with inflammation. Lymphoid tissue degenerates in adulthood. Therefore, tonsil hypertrophy is much more prevalent, characteristic [3–5], and causative to obstructive sleep-disordered breathing (SDB) in children [6]. Also in children, the tonsil can kindle infections that may give rise to acute or chronic tonsillitis [7]. Symptoms of tonsillitis include sore throat, fever, enlargement of the tonsil, difficult swallowing, and swollen lymph nodes around the neck. Recurrent tonsillitis (RI) is diagnosed when tonsillitis relapses as sympomized by ≥7 episodes of throat infection (well-documented, clinically important, adequately treated) in the preceding year, or ≥ 5 episodes in each of the preceding 2 years, or ≥ 3 episodes in each of the preceding 3 years [8].

Tonsillar disease is routinely treated with antibiotics or tonsillectomy [9]. Tonsillectomy is most recommended for children who meet the standards of the AAO-HNS tonsillectomy guidelines [10], but controversy still exists in the indications and the benefits/harms after eliminating this immune organ [11]. Although numerous studies have been carried out to explore the bacteriology in the tonsil, the underlying pathogenic mechanism of tonsil hypertrophy has not been fully understood.

Tonsil hypertrophy exhibits varying clinical manifestations, either as simple hypertrophy or hypertrophy accompanied with recurrent inflammation. These different clinical manifestations suggest that tonsillar hypertrophy might be regulated by diverse immune and/or inflammatory mechanisms. But so far, there have been few reports analyzing these differences. This study, therefore, aimed to demonstrate the differences in innate immune and inflammatory responses in tonsils with various clinical manifestations, which will refresh our understanding of the pathology of tonsil hypertrophy and improve related clinical prevention and treatment.

## Methods

### Human tissue samples

This study was reviewed and approved by the institutional review board. Informed consent was obtained from the legal caregiver of each participant. Before surgery, the children who underwent tonsillectomy for either SDB or RI were identified. The diagnosis of SDB was established by a spectrum of breathing disorders during sleep, habitual and loud snoring, and upper airway obstruction [6]. RI was clinically diagnosed if the patient had a history of tonsillitis and sore throat (≥3 infections per year in 3 years) that failed to respond to antibiotics. The degree of tonsil hypertrophy was scored from 1+ to 4+ according to the percentage of decreased pharyngeal lumen diameter [12–14]: 1+, 0–25%; 2+, 26–50%; 3+, 51–75% and 4+, 76–100%. Tonsil hypertrophy was scored 3–4+ preoperatively [15].

Patients advised to undergo tonsillectomy (TE) in accordance with the AAO-HNS tonsillectomy guidelines [10], were recruited into the study. Consecutive patients were divided into three groups according to the clinical manifestations of their tonsils: 1) control group; 2) tonsil hypertrophy (TH) group; and 3) tonsil hypertrophy combined with recurrent infection (TH + RI) group. Included were those [1]: aged 2 to 13 years [2]; exhibiting significant symptoms of snoring that disrupted the quality of sleep or daily activities; and [3] who planned to undergo adenoidectomy and tonsillectomy. Excluded were those who displayed [1]: symptoms of acute infection at the time of the surgery, and received antibiotic therapy within the previous 4 weeks [2]; congenital anomalies including clef palate, Down syndrome, congenital heart disease, and craniofacial anomalies [3]; major systemic disease, such as diabetes, nephrotic disease, autoimmune disorders, immunodeficiency, malignancy, and other chronic illnesses; or [4] known allergic conditions such as asthma, allergic rhinitis, or history of allergies. As we defined that tonsils with hypertrophy score < 3 were not hypertrophic, the patients with SDB and adenoid hypertrophy, and no tonsillar hypertrophy or inflammation were collected as controls. A total of 45 patients were included in our study, including 20 patients in control group, 10 patients in TH group, and 15 patients in TH + RI group.

TE was conducted using plasma wand with a high-frequency electrosurgery system (Coblator II, ArthroCare Corporation, Austin, TX, USA). TE procedures were performed by surgeons with ≥5 years’ operating experience. The tonsils were completely removed during TE. The tissues that were not required for pathological examination were immediately collected and delivered into icy phosphate buffered saline (PBS) plus antibiotics in a − 70 °C refrigerator within half an hour after excision under aseptic conditions.

### Histopathological examination

Tonsil tissues were fixed in 4% paraformaldehyde (PFA) for 16 h, then paraffin-embedded. The sections (5 μm thickness) were prepared and stained with Masson or hematoxylin-eosin (H&E) and analyzed under the light microscope.

### Real time quantitative PCR (qRT-PCR)

Total RNA was isolated from the tonsil using the Trizol reagent (Takara Bio Inc., Japan) following the manufacturer’s instructions, and reverse-transcribed using reverse transcription kit (Takara Bio Inc., Japan). qRT-PCR was performed using SYBR Green master mix (Vazyme) and 7500 Real-Time PCR System (Applied Biosystems, Foster City, CA, USA). Primers used for qRT-PCR are shown in Table 1. Cycling conditions were 95 °C for 10 min, followed by 40 cycles of 95 °C for 15 s and 60 °C for 1 min. The amount of mRNA for each gene was standardized with the internal control GAPDH, and calculated using the comparative cycle threshold (ΔΔCt) method.

**Table 1:** Primers used for qRT-PCR

Primer	Forward (5′ → 3′)	Reverse(5′ → 3′)
VP1	AGTCCTCCCACGTCACTCAC	TGATCACTAGGGTGGCATCA
IFN-α	GCCTCGCCCTTTGCTTTACT	CTGTGGGTCTCAGGGAGATCA
RIG-I	CTGGACCCTACCTACATCCTG	GGCATCCAAAAAGCCACGG
MAVS	CAGGCCGAGCCTATCATCTG	GGGCTTTGAGCTAGTTGGCA
NLRP3	CGTGAGTCCCATTAAGATGGAGT	CCCGACAGTGGATATAGAACAGA
TLR1	TGAACCTCAAGCACTTGGACC	CCCATAAGTCTCTCCTAAGACCA
TLR2	ATCCTCCAATCAGGCTTCTCT	GGACAGGTCAAGGCTTTTTACA
TLR3	TTGCCTTGTATCTACTTTTGGGG	TCAACACTGTTATGTTTGTGGGT
TLR4	AGACCTGTCCCTGAACCCTAT	CGATGGACTTCTAAACCAGCCA
TLR5	TCCCTGAACTCACGAGTCTTT	GGTTGTCAAGTCCGTAAAATGC
TLR7	TCGTGGACTGCACAGACAAG	GGTATGTGGTTAATGGTGAGGGT
TNF-α	CCTCTCTCTAATCAGCCCTCTG	GAGGACCTGGGAGTAGATGAG
IL-6	ACTCACCTCTTCAGAACGAATTG	CCATCTTTGGAAGGTTCAGGTTG
IL-1β	ATGATGGCTTATTACAGTGGCAA	GTCGGAGATTCGTAGCTGGA
NF-κB	AACAGAGAGGATTTCGTTTCCG	TTTGACCTGAGGGTAAGACTTCT
IL-7	TTCCTCCCCTGATCCTTGTTC	CTTGCGAGCAGCACGGAATA
IL-10	GACTTTAAGGGTTACCTGGGTTG	TCACATGCGCCTTGATGTCTG
IL-12A	CCTTGCACTTCTGAAGAGATTGA	ACAGGGCCATCATAAAAGAGGT
IL-17A	AGATTACTACAACCGATCCACCT	GGGGACAGAGTTCATGTGGTA
IL-18	TCTTCATTGACCAAGGAAATCGG	TCCGGGGTGCATTATCTCTAC
COX2	GAATCATTCACCAGGCAAATT	TCTGTACTGCGGGTGGAACA
EP1	ACCTTCTTTGGCGGCTCTC	CCAACACCAGCATTGGGCT
EP2	GCTCCTTGCCTTTCACGATTT	AGGATGGCAAAGACCCAAGG
EP3	AGCTTATGGGGATCATGTGC	TTTCTGCTTCTCCGTGTGTG
EP4	CGCTCGTGGTGCGAGTATT	AGGGGTCTAGGATGGGGTTC
GAPDH	CGCTCTCTGCTCCTCCTGTT	CATGGGTGGAATCATATTGG

### ELISA assay

The PGE2 levels in tonsils were determined by the ELISA kit (Elabscience, Wuhan, China) according to the manufacturer’s instructions.

### Statistical analysis

All the data were presented as mean ± SEM. GraphPad Prism version 5.0 (GraphPad Software, La Jolla, CA, USA) software was used for statistical analyses, including one-way analysis of variance (ANOVA) for multiple comparisons. An unpaired Student’s *t*-test was also performed to determine any significant difference between two groups. *P* < 0.05 was considered statistically significant.

## Results

### Pathological features of tonsils in each group

**Fig. 1:**
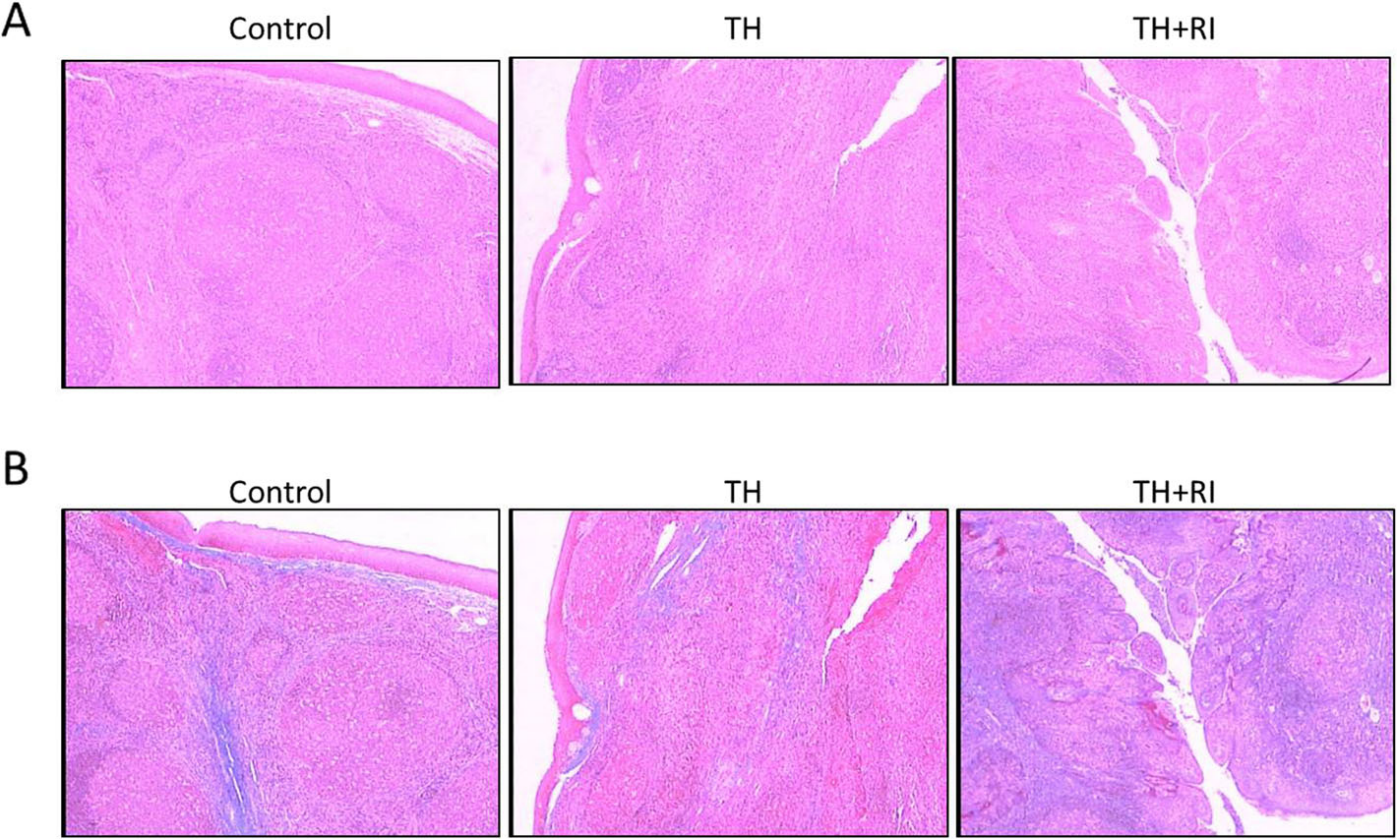
Pathological features of tonsils in each group. (a) Tonsil sections were stained with H&E and evaluated by microscope (× 200). (b) Tonsil sections were stained with Masson and evaluated by microscope (× 200)

H&E staining showed that the tonsils in the control group were composed of lymphoid tissues, superficial squamous epithelium, and some lymphoid nodules. The tonsils in TH group showed lymphoid hyperplasia, increased lymphoid follicles, and enlarged germinal centers. Besides these three changes, the tonsils in TH + RI group showed the epithelium with papillary hyperplasia or high-level infiltration of inflammatory cells (Fig. 1a). Masson staining showed that the tonsils in TH + RI group had developed more significant interstitial fibrosis, compared with the other two groups (Fig. 1b).

### Viral infection and innate immune response in each group

**Fig. 2:**
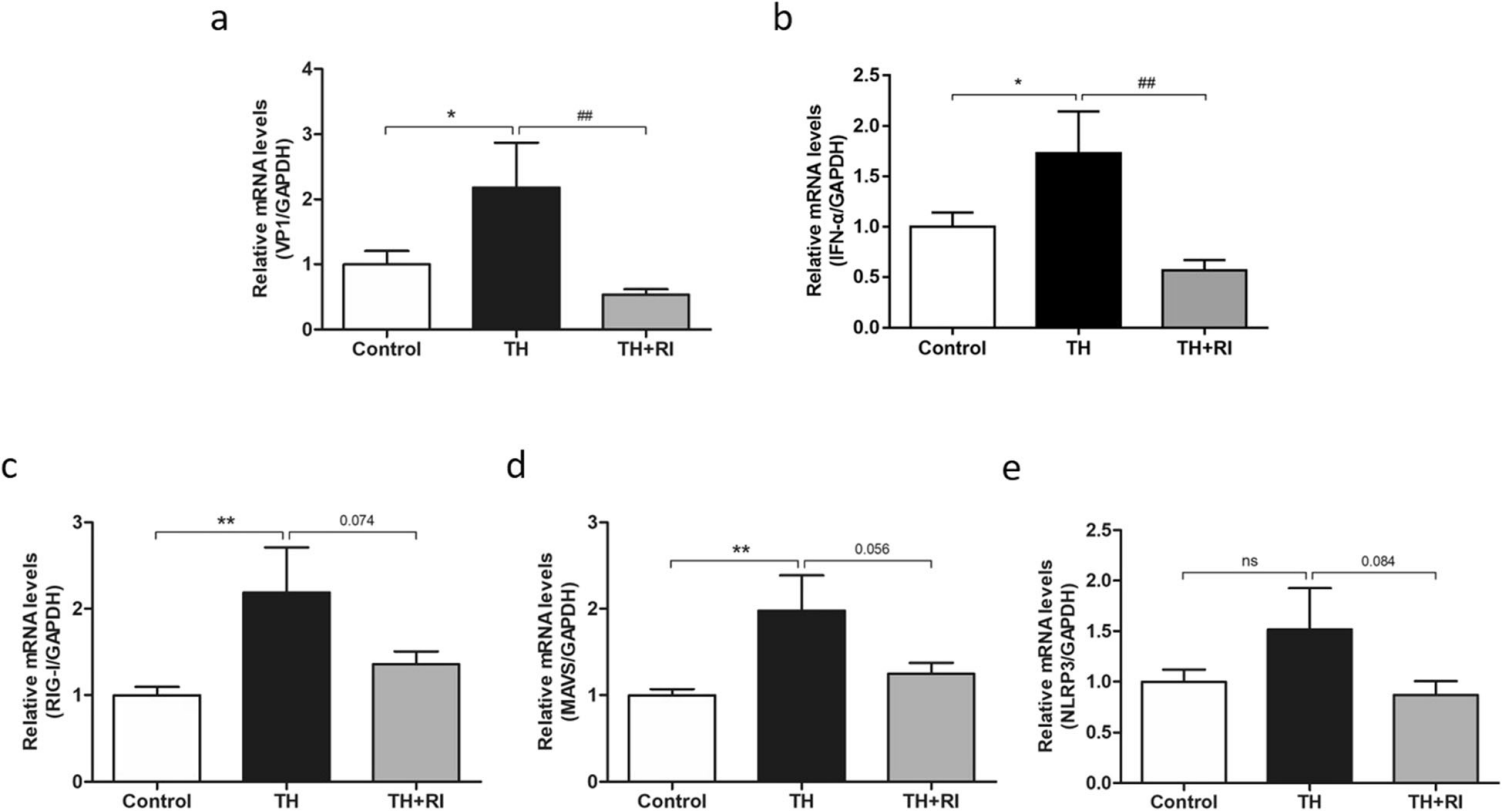
Viral infection and innate immune response in each group. (a-e) mRNA expressions of VP1, IFN-α, RIG-I, MAVS and NLRP3 in the tonsils were determined by qRT-PCR. Data were expressed as mean ± SEM. *n* = 20 in control group; *n* = 10 in TH group; *n* = 15 in TH + RI group.*P < 0.05 compared to the control group; **P < 0.01 compared to the control group; ^##^*P* < 0.01compared to the TH group; ns (no significance) compared to the control group

With qRT-PCR, we found that the expression levels of VP1 and IFN-α in TH group were significantly higher than that in the control group, but that in TH + RI group was significantly lower than that in the TH group (Fig. 2a&b). We next examined the pathways involved in innate immune response caused by the virus. We found that the mRNA levels of RIG-I and MAVS in TH group were significantly higher than that in the control group, while the mRNA levels of RIG-I and MAVS in TH + RI group were lower than that in TH group (Fig. 2c&d). The expression profiles of RIG-I and MAVS were consistent with that of VP1 and TNF-α. NLRP3, a component of the innate immune system, was also detected in each group. The expression of NLRP3 in TH group was up-regulated, compared with the control group, while the expression level of NLRP3 in TH + RI group significantly decreased (Fig. 2e). These results indicate that the innate immune response in the tonsil was more active in TH group than in TH + RI group.

### Expression of toll-like receptors (TLRs) in each group

**Fig. 3:**
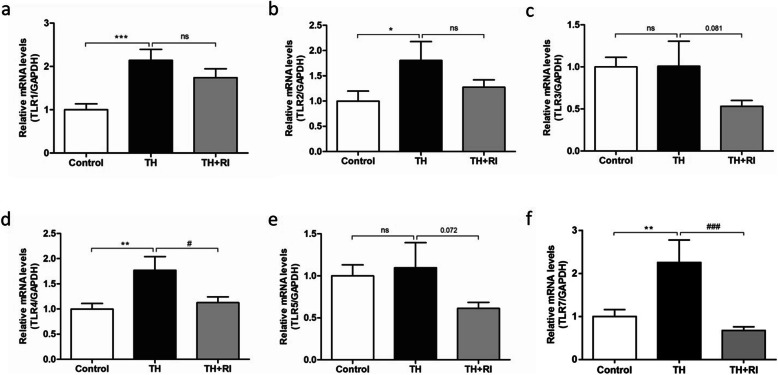
Expression of TLRs in each group. (a-f) mRNA expressions of TLR1, TLR2, TLR3, TLR4, TLR5 and TLR7 in the tonsils were determined by qRT-PCR. Data were expressed as mean ± SEM. *n* = 20 in control group; *n* = 10 in TH group; *n* = 15 in TH + RI group. ^*^P < 0.05 compared to the control group; ^**^P < 0.01 compared to the control group; ^***^P < 0.001 compared to the control group; ^#^*P* < 0.05 compared to the TH group; ^###^*P* < 0.001 compared to the TH group; ns (no significance) compared to the control group or TH group

TLRs and their signaling play a crucial role in innate immune response [16]. Therefore, we examined the expression of TLRs. Compared with the control group, the expression levels of TLR1, TLR2, TLR4 and TLR7 in TH group were significantly up-regulated (Fig. 3a, b, d&f). TLR3 and TLR5 in TH group did not change significantly (Fig. 3c&e). The expression levels in TLRs (especially TLR4 and TLR7) in TH + RI group were significantly decreased, compared with TH group (Fig. 3d&f). These results indicate that TLR1, TLR2, TLR4, TLR7 may be involved in the activation of the innate immune response in TH group.

### Inflammatory response in each group of tonsils

**Fig. 4:**
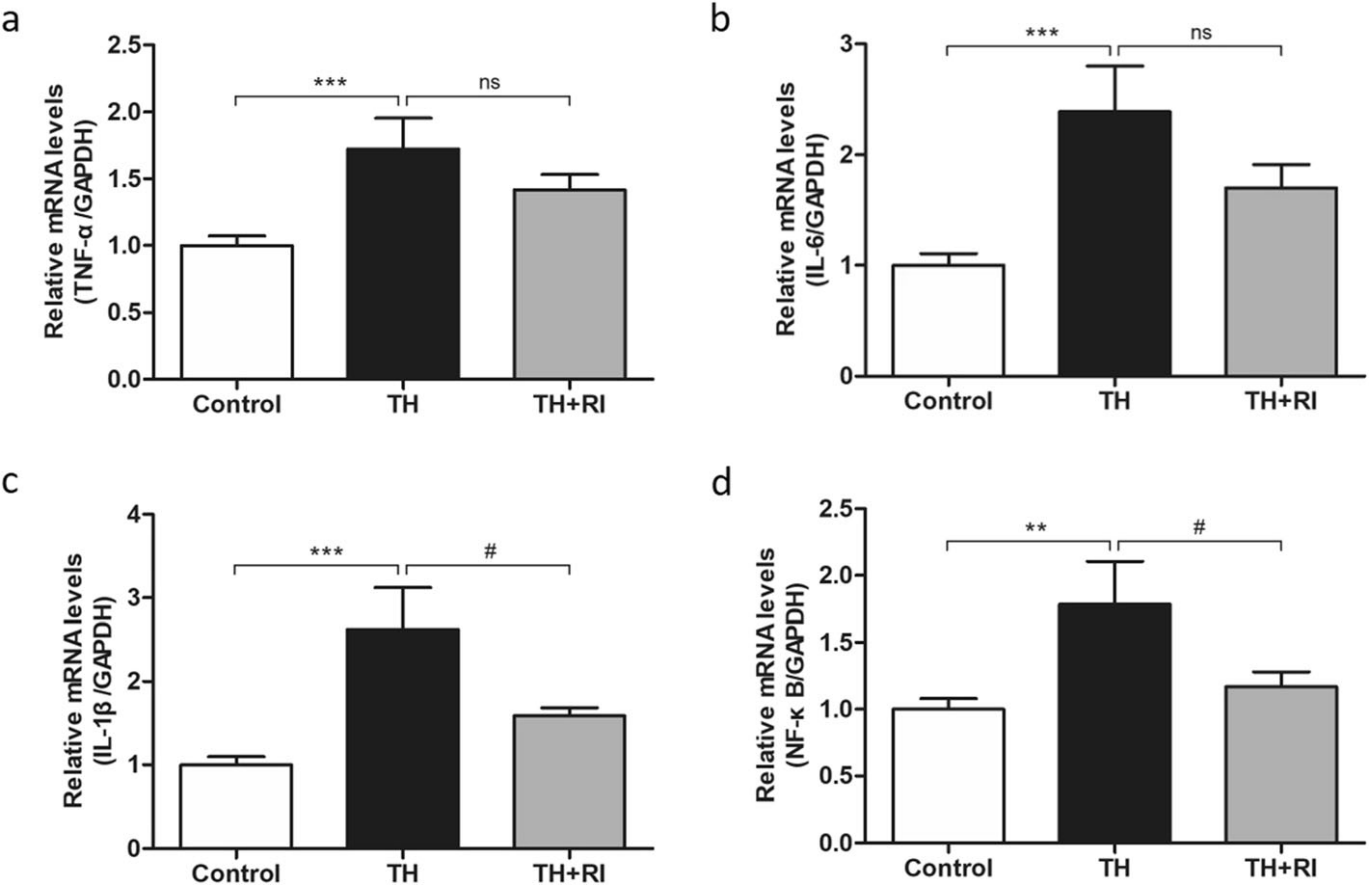
Inflammatory response in each group. (a-d) mRNA expressions of TNF-α, IL-6, IL-1β and NF-κB in the tonsils were determined by qRT-PCR. Data were expressed as mean ± SEM. n = 20 in control group; n = 10 in TH group; n = 15 in TH + RI group. ^**^P < 0.01 compared to the control group; ^***^P < 0.001 compared to the control group; ^#^*P* < 0.05 compared to the TH group; ns (no significance) compared to the TH group

**Fig. 5:**
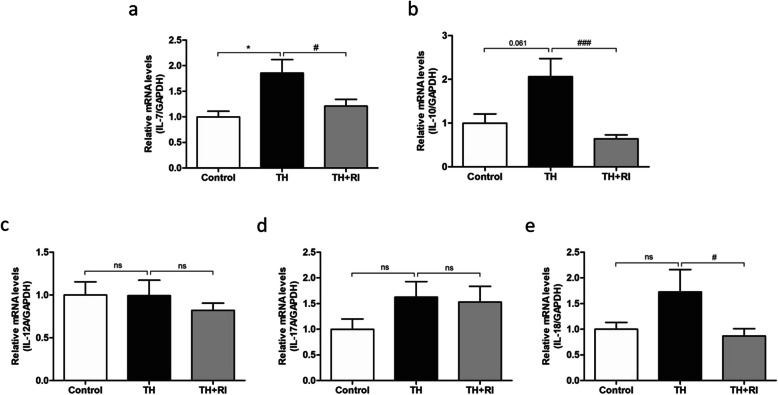
Expression of some other inflammatory factors in each group. (a-e) mRNA expressions of IL-7, IL-10, IL-12A, IL-17A and IL-18 in the tonsils were determined by qRT-PCR. Data were expressed as mean ± SEM. n = 20 in control group; n = 10 in TH group; n = 15 in TH + RI group. ^*^P < 0.05 compared to the control group; ^#^*P* < 0.05compared to the TH group; ^###^*P* < 0.001 compared to the TH group; ns (no significance) compared to the control group or TH group

To assess the inflammatory response, we examined the mRNA levels of TNF-α, IL-6, IL-1β and NF-κB. The results showed that the expression levels of these four inflammatory factors in TH group were significantly higher than those in the control group, while these inflammatory factors in TH + RI group were increased to varying degrees, compared with the control group, but this increase was less obvious than that in the TH group. In particular, the mRNA levels of IL-1β and NF-κB were significantly lower than those in TH group (Fig. 4a-d). These results suggested that the inflammatory response in TH group was stronger than that in TH + RI group. We also tested the mRNA levels of IL-7, IL-10, IL-12A, IL-17A and IL-18. The results showed that IL-7 and IL-10 levels in TH group had an increasing trend, compared with the control group, and the levels in TH + RI group were significantly lower than those in TH group (Fig. 5a&b). The levels of IL-12A, IL-17A and IL-18 in TH group were not significantly different from those in the control group. IL-12A and IL-17A levels in TH + RI group were not significantly different from those in TH group (Fig. 5c-e).

### Expression of COX-2, PGE2 and its receptors in each group

**Fig. 6:**
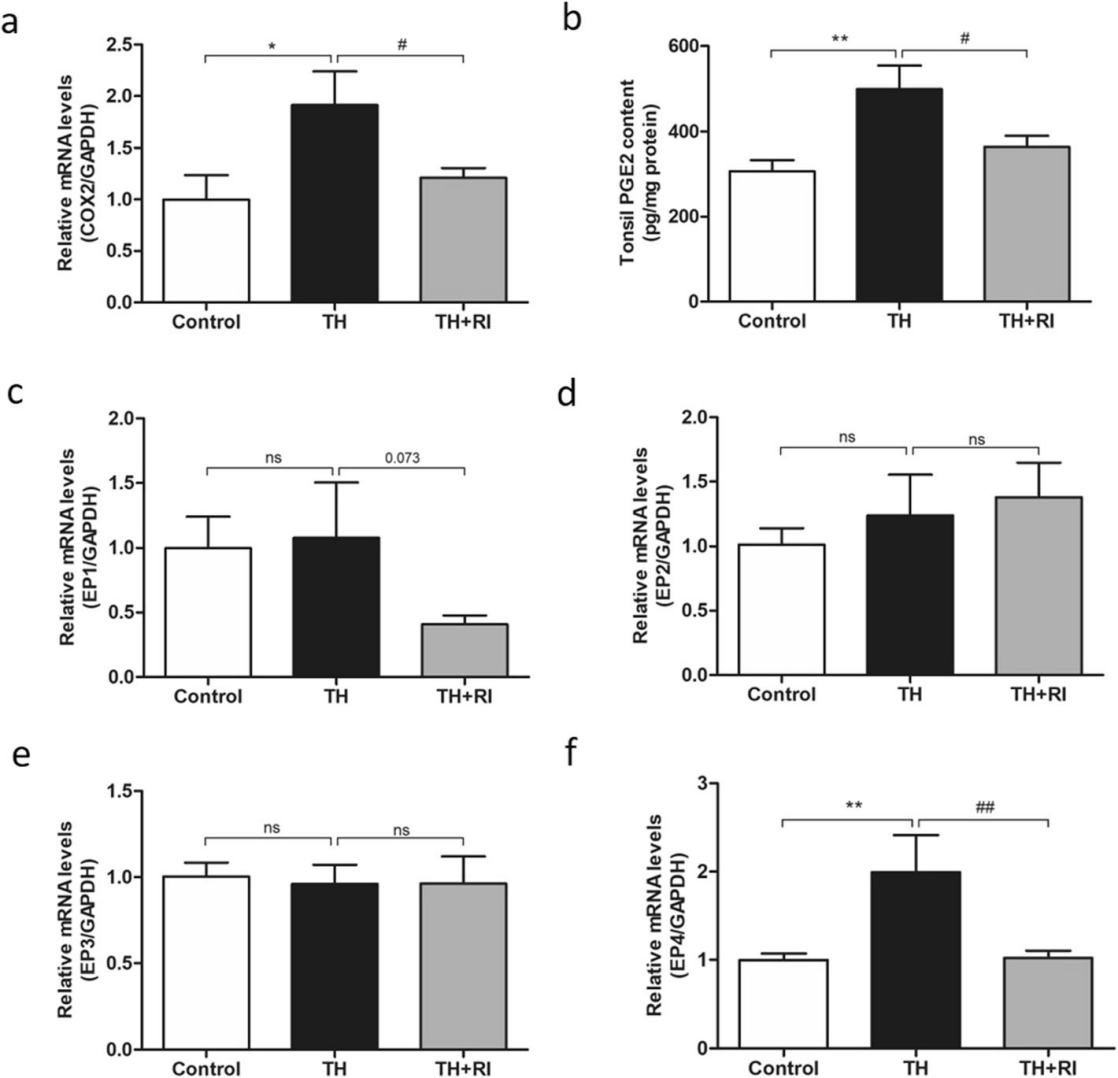
The changes of COX-2, PGE2 and its receptors in each group. (a) mRNA expression of COX-2 in the tonsil was determined by qRT-PCR. (b) PGE2 content in the tonsil was determined by ELISA. (c-f) mRNA expression of EP1, EP2, EP3 and EP4 in the tonsil was determined by qRT-PCR. Data were expressed as mean ± SEM. n = 20 in control group; n = 10 in TH group; n = 15 in TH + RI group. ^*^P < 0.05 compared to the control group; ^**^P < 0.01 compared to the control group; ^#^*P* < 0.05 compared to the TH group; ^##^*P* < 0.01 compared to the TH group; ns (no significance) compared to the control group or TH group

Cyclooxygenase (COX) catalyzes Cox (bis-oxygenase) reaction in which arachidonic acid is converted into PGG2 and then PGG2 is reduced by two electrons into PGH2, a precursor to all prostanoids, including PGE2, PGD2, PGF2, PGI2, and thromboxane. Prostaglandins are critical mediators in inflammation [17]. Through assessing the expression of COX-2, PGE2 and its receptors, we teased out the prostaglandin-related signaling pathways in the tonsil inflammatory response. The results of qRT-PCR showed that the expression of COX-2 in TH group was significantly higher than that in the control group. The expression level of COX-2 in TH + RI group was not significantly higher than that in the control group, but significantly lower than that in TH group (Fig. 6a). The ELISA results also showed that the level of PGE2 in TH group was significantly up-regulated and higher than that in TH + RI group (Fig. 6b). We then tested the receptors for PGE2, including EP1, EP2, EP3 and EP4, by qPCR. The results showed that only the expression of EP4 was significantly changed in the three groups. The expression of EP4 in TH group was significantly higher than that in the control group, while that in TH + RI group was close to that in the control group (Fig. 6c-f). These results suggest that the COX-2/PGE2/EP4 axis may be involved in the tonsillar inflammatory response.

## Discussion

Tonsil hypertrophy is a common otolaryngological disease, affecting 5–27% of children with primary snoring [18] and 10–12% of SDB children aged 2–8 years [6]. The etiology of hypertrophy in tonsillar lymphoid tissue remains unknown.

In the present study, the high expression of VP1 in the simple hypertrophic tonsils indicated that tonsil hypertrophy may be caused by viral infection. The innate immune system serves as the first line of defense against various microorganisms. Recent studies have shown various molecules playing critical roles in innate antiviral immune response [19–21]. The RIG-I-MAVS-mediated antiviral immune response is triggered in the host upon the invasion of pathogens. RIG-I recognizes viral RNA, undergoes conformational changes, and releases its CARDs domain. E3 ubiquitin ligase TRIM25 is recruited to catalyze the ubiquitination of K63 in RIG-I, and then binds to the downstream linker protein MAVS to transmit anti-transfer virus signal [22]. NLRP3 is an innate immune signaling receptor. Once activated, it initiates caspase1-mediated proteolytic activation of the IL-1β family of cytokines, and induces an inflammatory response [23–25]. The expression of these innate immune factors, including RIG-I, MAVS and NLRP3, may be up-regulated by viral infections in simple hypertrophic tonsils.

The innate immune system relies on TLRs to recognize and bind pathogen-associated molecules. It has been demonstrated that TLRs recognize viruses and initiate a series of cellular antiviral responses via intracellular signaling pathways [26, 27]. Our results showed that TLR4 and TLR7 were involved in virus-geared innate immune response in simple hypertrophic tonsils. The activation of TLR initiates downstream signaling cascades (like MAPK or NF-κB) and consequent proinflammatory response [28, 29]. TLR7 can activate NF-κB to mediate the immune inflammatory response. Consistent with their performance in innate immune response, IFN-α, NF-κB, TNF-α, IL-1β and IL-7 showed up-regulated expression in the simple hypertrophic tonsils. The high levels of TNF-α and IL-6 in TH group reflected that monocyte-macrophage transformation was enhanced, thus inducing the activation and proliferation of endothelial cells and fibroblasts. In this process, immunologically active tissue can be gradually replaced by fibrotic tissue [30]. This may be the reason why TH group exhibited stronger inflammation than TH + RI group.

Over activation of TLR4 signaling pathway can cause over expression of various inflammatory mediators, including COX-2 [31]. Recent studies have verified the contribution of inducible COX-2 and its downstream prostaglandin signaling pathways in modulating inflammatory responses [32–34]. PGE2 is generated through a sequential enzyme cascade of COX/PGE2 synthases (PGES) [35], and mediated via four different G-protein-coupled PGE receptors (e.g. EP1, EP2, EP3, and EP4) [36]. PGE2 appears to facilitate the function of helper T cells mainly via EP4 in vivo, and modulate the pathogenesis of various chronic inflammation and autoimmune diseases [37]. In the simple hypertrophic tonsils, the up-regulation of COX2, PGE2 and EP4 indicates that this pathway may be responsible for the inflammatory response and formation of fibroblasts. Whether this pathway can be used as a target in the treatment of tonsil hypertrophy requires further research.

Also, RI may lead to fibrosis of the tonsils and to fixation of the tonsil in its bed. The volume of the tonsils cannot be relied on to establish the diagnosis of tonsillitis, but to define its symptoms, such as SDB [38]. Different production of inflammatory and innate immune mediators suggested that tonsillar tissue hypertrophy may be regulated by divergent mechanisms and have different immunophenotype. Detecting these differences may help otolaryngologists to administer target treatments for hypertrophic tonsils. However, lacking data of RI is a limitation of our study, which should be resolved in our future work.

In summary, our results suggest that innate immune and inflammatory responses are more active in simple hypertrophic tonsils, rather than hypertrophic tonsils with recurrent inflammation. On the other hand, a local relative immune deficiency in the hypertrophic tonsils may be a causative factor for recurrent tonsillitis in TH + RI. These differences, together with the patient’s clinical manifestations, suggest that tonsillar hypertrophy might be regulated by diverse immune and/or inflammatory mechanism. Our findings provided a new understanding on the pathogenesis of tonsil hypertrophy, through which novel therapeutic strategies might be created.

## Data Availability

The datasets used and/or analyzed during the current study are available from the corresponding author on reasonable request.
